# Repairing spinal cord injuries: The most promising polymeric biomaterials and local drug delivery

**DOI:** 10.4103/NRR.NRR-D-25-00460

**Published:** 2025-10-30

**Authors:** Inês Martins, Cláudia Nunes, Diogo Trigo

**Affiliations:** 1 CICECO-Aveiro Institute of Materials, Department of Materials and Ceramic Engineering, University of Aveiro, Aveiro, Portugal; 2 iBiMED-Institute of Biomedicine, Department of Medical Sciences, University of Aveiro, Aveiro, Portugal

**Keywords:** drug delivery, polymers, recovery, regeneration, spinal cord injury

## Abstract

Spinal cord injury results in severe sensory and motor deficits, with a dramatic impact on lifespan and healthspan, due to the limited regenerative capacity of the central nervous system. Following the initial injury, glial scarring and extracellular matrix barriers prevent axonal regeneration, while secondary damage exacerbates inflammation and tissue loss. The gold standard treatment in the last decades is acute surgery and systemic drug administration. New technologies have been developed, such as stem cell therapies, or the combinatory use of biomaterials, nonetheless still offering limited recovery. Successful therapies must act directly on neurons, activating regenerative pathways, while simultaneously promoting a pro-regenerative environment. However, polymeric materials have, in recent years, shown a regenerative potential through a series of different processes, from promoting neural regeneration by supporting tissue repair and reducing inflammation, to local drug delivery mechanisms. In this work, we systematize the state-of-the-art, exploring recent advancements in polymer-based therapies for spinal cord injury, discussing their characteristics, limitations, and promising future applications.

## Introduction

Bodily functions and activities are controlled by the central nervous system (CNS). The brain coordinates both voluntary and involuntary movements, while the spinal cord channels signals between the brain and the periphery and regulates reflexes.

**Figure 1:**
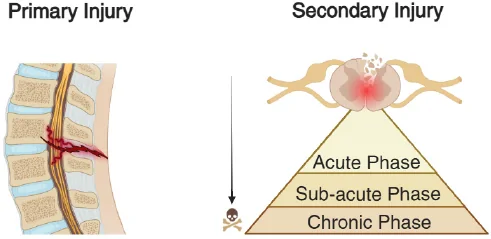
Representation of primary and secondary injuries of spinal cord injury. Primary injury illustration consists of a side view of vertebral column, spinal cord, intervertebral discs, with an injury that affects the spinal cord directly, to understand the primary injury is the first stage of spinal cord injury, right after the injury happens. Secondary injury illustration consists of a top view of the spinal cord lesion, with tissue loss, and presents a pyramid that comes out of this illustration. Going down the pyramid, the severity of the injury and the risk of mortality increase, which is why it has an arrow with a skull pointing downwards. The different levels of the pyramid represent the 3 stages of secondary injury. Created with BioRender.com.

Due to the poor regenerative capacity of the adult CNS, spinal cord injury (SCI) frequently results in severe sensory and motor deficits. An impermeable barrier forms immediately after injury, due to the inhibitory environment of the lesion, the development of a glial scar, and the accumulation of proteoglycans from the extracellular matrix (ECM), preventing axonal regeneration. Furthermore, it causes a chain reaction of cellular and metabolic processes that result in a series of reactive alterations, including inflammation, hemorrhagic necrosis, edema, and demyelination, causing cell death, vascular destruction, scarring, and axonopathy with loss of functional connections below the central lesion site (Pêgo et al., 2012). Long-term outcomes of SCI are closely related to three main topics: the level of injury, the severity of the primary injury, and progression of the secondary injury (**Figure 1**). In the primary injury, bone, disc, or ligament debris bruise or damage the spinal tissue. The degree of initial damage and length of spinal compression determine the severity of SCI. The primary injury triggers a secondary injury, which results in chemical and mechanical damage to spinal tissues, leads to neuronal excitotoxicity mediated by glutamate, and increases the levels of reactive oxygen species. Sub-acute secondary injury leads to the last phase, chronic secondary injury, characterized by phenomena such as axonal dieback and maturation of glial scar (Anjum et al., 2020).

SCI has numerous socioeconomic repercussions on both patients and society, leading among them is the overcrowding of the hospital system (Alizadeh et al., 2019). In 2019, the anticipated global incidence was between 250,000 and 500,000 individuals every year (Safdarian et al., 2023), with over 1 million years of healthy life lost over a 44-year span, and 445,911 disability-adjusted life years in a single year (Hall et al., 2019).

This condition causes chronic pain (Huang et al., 2019) and whole or partial loss of sensation and mobility caudal to the injury site, which, depending on size and location, can result in paraplegia or tetraplegia (Alizadeh et al., 2019). Besides severe sensory and motor deficits, SCI is marked by a significant challenge to full recovery due to the reduced growth capacity of mature neurons in the spinal cord (Ding et al., 2022), the lack of plasticity, and the global limited regenerative capacity of the central nervous system (Pêgo et al., 2012; Ashammakhi et al., 2019). Apart from causing reduced motor function, such as paralysis, this disorder can also lead to numerous other severe health complications such as breathing, urogenital, circulatory, and psychological disorders (Yamazaki et al., 2020). These issues are summarized in **Table 1**.

**Table 1:** Representation of systemic medical problems caused by spinal cord injury

Type of health problem	Examples	References
Motor	Paralysis; spasticity	Rekand and Merete Hagen, 2012; Debenham et al., 2024
Sensory	Analgesia; allodynia	M’Dahoma et al., 2014; Yamazaki et al., 2020
Circulatory	Bradycardia; arrhythmia; postural hypotension; deep venous thrombosis; pulmonary embolism	Sidorov et al., 2008; Hector et al., 2013; Mackiewicz-Milewska et al., 2016; Ong et al., 2023
Urogenital	Dysuria; urinary tract infection; sexual dysfunction	Amaral et al., 2019; Zizzo et al., 2022; Yu et al., 2024
Digestive	Stomach-duodenal ulcer; ileus; defecation disorder	Kewalramani, 1979; Vallès et al., 2006; Baumann et al., 2009
Mental	Depression; suicide	Cao et al., 2014; Khazaeipour et al., 2015
Others	Thermoregulatory dysfunction; pressure ulcer; heterotopic ossification	Regan et al., 2009; Handrakis et al., 2017; Schincariol et al., 2023

## Search Strategy

A comprehensive search was conducted in the following databases: PubMed (via NIH), Scopus (via Elsevier), SpringerNature Link, Research Gate, and Wiley Online Library.

Searches were performed from September 2024 to June 2025, and covered literature published from 2016 to 2025 except information of general knowledge or information already well established in the literature.

The search strategy included a combination of keywords and subject headings related to “spinal cord injury,” “neuronal regeneration,” and all the polymers listed during this review paper, adapted for each database. Synonyms and related terms were identified through preliminary literature reviews. Boolean operators and abbreviations were used to refine the search. No specific filter was applied. Additionally, reference lists of relevant reviews and articles were manually screened to identify further studies.

Moreover, a comprehensive search was conducted on clinicaltrials.gov, combining the condition/disease “spinal cord injury” with the different explored materials.

## Mechanisms of Neural Regeneration

Neural regeneration represents a critical area of research with profound implications for the treatment of SCI.

Processes of CNS neural regeneration are intricate and quite different from peripheric ones, since CNS lesions frequently result in severe disability, while peripheral nerves frequently heal after injury. While peripheric neurons have a certain regenerative ability, the main hurdle with SCI is the limited regenerative capacity of central neurons, as regeneration is inhibited by neuron-intrinsic mechanisms and by the formation of the glial scar, which acts as a physical barrier and creates an inhibitory environment (Silver and Miller, 2004; Nagappan et al., 2020). Research has explored promoting axonal regeneration to re-establish the circuitry for functional recovery, but only a fraction of damaged neurons regenerate, achieving a short elongation. Another confounding factor is the requirement of adequate distribution along the neuron for axonal outgrowth and regeneration (Han et al., 2016), but axotomy results in a localized energy crisis due to mitochondrial membrane depolarization (Cartoni et al., 2016), indicating that energy deficiency alone could possibly hinder regeneration.

Several mechanisms can modulate neural regeneration after injury, such alterations of gene expression and calcium signaling pathways; the growth cone collapses after injury, but the influx of Ca^2+^ can activate signaling pathways promoting cytoskeletal rearrangement, essential for growth cone motility and directionality; blocking the myelin-associated inhibitors action and enhancing other positive signaling pathways between neurons and glial cells (Mahar and Cavalli, 2018).

Since regeneration does not occur naturally in the central nervous system, treatments that promote this process are required, and current research is focused on finding the best way to successfully regenerate tissue and restore lost functions.

## Mainstream Treatments for Spinal Cord Injury

The most common treatments following SCI are surgery, stem cell and drug-targeted therapies, biomaterials, tissue engineering, and regenerative medicine. Although new pharmacological tools have been developed in recent years, the challenge is the form of administration (as discussed below). Any promising drug must tackle the tissue complexity of the spinal cord environment and affect various cell types to effectively promote neuroregeneration (Goncalves et al., 2015), remyelination (Goncalves et al., 2019), and minimize scar formation (Goncalves et al., 2018). Current pharmaceutical treatments still often fail to control pain, and surgery is normally the first line of treatment (Feng et al., 2023). However, several new pharmacological treatments have also been explored for SCI. For instance, the chronic administration of the antibiotic dapsone was recently shown to decrease the inflammation and promote functional recovery (Calderón-Estrella et al., 2023), while the quinolizidine alkaloid aloperine was reported to reduce tissue damage, apoptosis, oxidative stress, and inflammation, leading to improved locomotor function (Okutan et al., 2025), in rat lesion models.

Different techniques for neuroprotection and neuroregeneration have been explored, with varying degrees of success. Removal of bone fragments or foreign objects, physical stabilization of the vertebral column, and spinal decompression are all examples of stabilization procedures (Ashammakhi et al., 2019). Additionally, cerebrospinal fluid drainage (Kwon et al., 2009) and hypothermia (Tracy et al., 2015) are two anti-inflammatory non-drug approaches that showed early promise, while anti-inflammatory medication delivery, blood pressure augmentation, and early surgical decompression also decrease acute problems (Ahuja et al., 2017). However, since then, progress has encountered roadblocks.

Spinal cord stimulation, originally developed for temporary pain management, is currently applied up to months after injury (Marco, 2022), with interesting results in bone and muscle health, helping to regain some motor control, and providing particularly effective pain relief (by yet unclear mechanisms), lowering medication consumption and enhancing quality of life (Huang et al., 2019). Neuromodulation is an emerging tool, showing promising results for SCI treatment (Abraham et al., 2023), including in clinical trials (Rowald et al., 2022).

Nonetheless, despite extensive research on regenerative treatments over the past few decades, SCI management remains particularly challenging due to a lack of procedures that can restore lost tissue function. Recent advances in cell-based therapies, biomolecules, and biomaterials have led to the development of therapeutic strategies for neuroregeneration with promising *in vivo* results (Ashammakhi et al., 2019). In addition, the potential of biomaterials to promote regeneration, by treating and repairing tissues or delivering therapeutic agents, remains a major focus of ongoing research.

These materials have been explored in different forms and applications; an ideal theoretical material would be an implantable one that could be kept in place for several months, directing regeneration and stopping chronic inflammation, degrading as the tissue regenerates (Ashammakhi et al., 2019). No such therapy is currently used in regular clinical practice; however, various types of bioresorbable polymer scaffolds have been tested in animal models, and even been implemented in a patient, demonstrating its therapeutic validity for an acutely contused spinal cord (Lu et al., 2022). Alternatively, polymers can be designed to remain *in situ*, not degrading, such as in the prosthetics field, but in most clinically relevant situations, a temporary removal might be required. Biodegradable biomaterials eliminate the need for follow-up surgeries, making them ideal for short-term use (Ashammakhi et al., 2019).

## Polymeric Materials for Spinal Cord Injury Recovery

**Figure 2:**
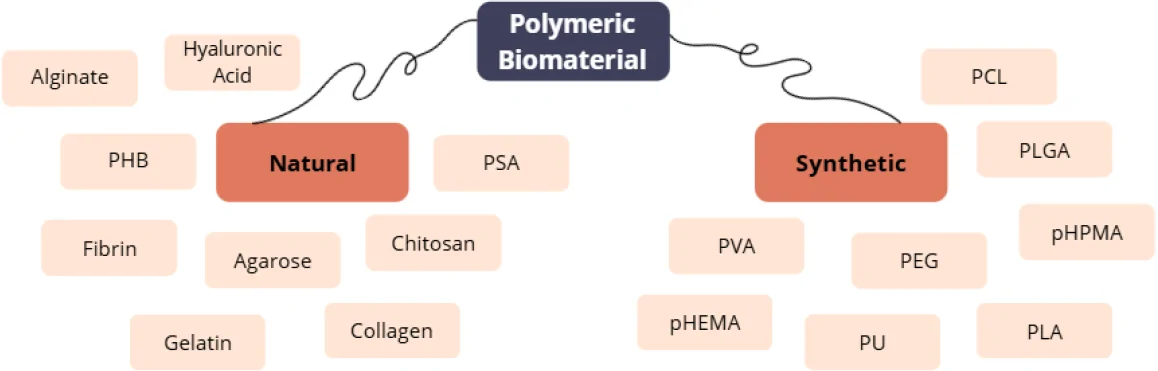
Polymers used to develop novel devices for spinal cord injury treatments. The figure represents a scheme of the most used materials for spinal cord injury, which are discussed throughout the article. Starting with the polymeric biomaterial, it can be natural or synthetic, and in each of these groups, the various polymers covered are presented. Abbreviations used are explained in the article. Created with Canva. PCL: Polycaprolactone; PEG: polyethylene glycol; PHB: polyhydroxybutyrate; pHEMA: poly(2-hydroxyethyl methacrylate); pHPMA: poly (N-(2-hydroxypropyl) methacrylamide); PLA: polylactic acid; PLGA: poly(lactic-co-glycolic acid); PSA: polysialic acid; PU: polyurethane; PVA: polyvinyl alcohol.

Both natural and synthetic materials are currently being explored for developing biocompatible devices, including micropatterned matrices and biocompatible scaffolds that support tissues and promote spinal cord regeneration (Ashammakhi et al., 2019; Lis-Bartos et al., 2020). Natural versus synthetic decision must predate device development (**Figure 2**).

The most frequently used natural polymers have low costs and are easy to obtain, such as alginate, agarose, chitosan, collagen, fibrin, gelatin, hyaluronic acid, polysialic acid (PSA), and polyhydroxybutyrate (PHB). The most common synthetic materials are usually synthetized ones and do not exist naturally. These polymers include polycaprolactone (PCL), polylactic acid (PLA), poly(lactic-co-glycolic acid) (PLGA), polyethylene glycol (PEG), poly(2-hydroxyethyl methacrylate) (pHEMA), poly (N-(2-hydroxypropyl) methacrylamide) (pHPMA), and polyvinyl alcohol (PVA) (Ashammakhi et al., 2019; Feng et al., 2023). **Table 2** summarizes the crucial points about each polymer used to treat SCI.

**Table 2:** Summary of origin, synthesis complexity, cost, and safety of each polymer

Polymer	Origin	Synthesis complexity	Cost	Reference
Alginate	Natural (brown algae)	Low-aqueous extraction	Low	Rahman et al., 2024
Agarose	Natural (red algae)	Low-hot water extract + purification techniques	Low	Jiang et al., 2023
Chitosan	Natural (crustaceans exoskeletons)	Moderate-alkaline extraction or bioextraction	Low	Muthu et al., 2021
Collagen	Natural (animal ECM)	High-acid/alkaline/enzyme hydrolysis, ultrasound-assisted	Medium	Lynn et al., 2004; Matinong et al., 2022
Gelatin	Natural (collagen denaturation)	Low-acid/basic/enzymatic hydrolysis of collagen	Medium	Rather et al., 2022
Fibrin	Natural (from fibrinogen)	Moderate-enzyme induced proteolysis	High	Pieters and Wolberg, 2019
Hyaluronic Acid	Natural (ECM)	High-tissue extraction or microbial fermentation	High	Serra et al., 2023
PSA	Natural (microbial)	High-fermentation or enzymatic polymerization	High	Liu et al., 2010
PHB	Natural (bacterial)	Moderate-microbial fermentation	High	Jha and Pandya, 2024; Radadiya et al., 2024
PCL	Synthetic	Moderate-ring opening polymerization	Low	Wang et al., 2023
PLA	Synthetic (lactic acid)	Moderate-polycondensation or ring opening polymerization	Low	Widjaja et al., 2023
PLGA	Synthetic (lactic and glycolic acids)	Moderate-ring opening polymerization	Medium	Little et al., 2021
PEG	Synthetic (ethylene oxide)	Moderate-anionic ring opening polymerization	Medium/High	Bento et al., 2024
pHEMA	Synthetic	Moderate-free radical polymerization	Low/Medium	Oyarce et al., 2020; Siddiqui et al., 2016
pHPMA	Synthetic	High-controlled radical polymerization (RAFT/ATRP)	High	Pan et al., 2019; Tucker et al., 2015; Wu et al., 2005
PVA	Synthetic	Low-hydrolysis of poly (vinyl acetate)	Low	Bercea, 2024

ECM: Extracellular matrix; PCL: polycaprolactone; PEG: polyethylene glycol; PHB: polyhydroxybutyrate; pHEMA: poly(2-hydroxyethyl methacrylate); pHPMA: poly (N-(2-hydroxypropyl) methacrylamide); PLA: polylactic acid; PLGA: poly(lactic-co-glycolic acid); PSA: polysialic acid; PVA: polyvinyl alcohol.

## Material Safety

When developing and using polymers for neural tissue regeneration in clinical settings, material safety is a crucial factor. In addition to being biocompatible, biomaterials must also have suitable degradation profiles, low immunogenicity, and no neurotoxicity because the central and peripheral nervous systems are extremely sensitive environments. Natural or synthetic polymers that are frequently studied for neural repair are typically chosen for their shown safety profiles in preclinical and clinical settings.

Because of their structural resemblance to native ECM, natural polymers are intrinsically biocompatible. Usually, the host tissue tolerates their degradation products as they are not harmful. However, the immunogenicity of these compounds may be impacted by their source and purification techniques. For instance, improperly purified chitosan or alginate may contain endotoxin or protein contaminations that can lead to inflammation and a decrease in biocompatibility (Ménard et al., 2010; Reay et al., 2023). The controlled and repeatable features of synthetic polymers are a benefit. Most of these substances are deemed safe for several biomedical applications, but their byproducts and rates of degradation are important factors. For example, PLA and PLGA degrade into lactic acids and lactic and glycolic acids, respectively (Chen et al., 2024), which might result in localized acidity due to the autocatalytic effect and may have an impact on the viability of cells (Lu et al., 2000; He et al., 2013). Therefore, to guarantee safety in clinical use, strict quality control and sterilizing procedures are necessary.

The synthesis of both natural and synthetic polymers entails intricate processes that demand exact regulation of reaction conditions and molecular design, as advanced polymerization methods combine multicomponent reactions with controlled polymerization to customize macromolecular structures with targeted functions. Natural polymers, also commonly known as biopolymers, are naturally derived, while synthetic polymers are produced through chemical reactions. On one hand, natural materials involve less hazardous chemistry but sometimes present challenges in purification and modification processes. On the other hand, producing synthetic polymers often requires complex chemical synthesis, specialized equipment, or even toxic reagents/conditions. Information on the several materials used for SCI treatment research can be found in **Table 2**.

## Natural Polymers

Natural macromolecules, found in nature and generally non-toxic, include proteins, lipids, polysaccharides, and nucleic acids. Natural polymers can be used to provide materials with biological activity and can incorporate natural tissue features, creating noncytotoxic hydrogels and scaffolds (Joyce et al., 2021).

### Alginate

Sodium alginate is a hydrophilic, linear, and anionic polysaccharide, typically extracted from brown algae, containing blocks of β-D-mannuronic acid (M) and α-L-guluronic acid (G) residues combined through 1,4-glycosidic linkages (Zhang et al., 2021; Adamiak and Sionkowska, 2023).

In addition to the interesting properties such as biodegradability, biocompatibility, and non-toxicity, alginate is easy to obtain at a considerably low cost. Due to these features, it has several applications in the biomedical field. Alginate-based materials are very common in tissue engineering (blood vessels, bone, cartilage, muscle, pancreas, liver, or nerve), wound healing, drug delivery, and *in vitro* cell culture (Lee and Mooney, 2012; Adamiak and Sionkowska, 2023). Additionally, it easily complexes in the presence of divalent cations (usually Ca^2+^) to form a gel (Lee and Mooney, 2012). The application of alginate hydrogels in regenerative medicine has been growing exponentially, showing significant and promising results in several *in vivo* models (Grijalvo et al., 2019).

The physicochemical characteristics, composition, mode of administration, and other variables influence alginate *in vivo* biological behavior (Zhang et al., 2021). These wound healing-promoting materials are particularly interesting for tissue engineering because of their capacity to integrate medications and cells: alginate scaffolds encapsulating neural stem cells (NSCs) inhibit inflammation and reduce lesion size, improving recovery in an *in vivo* model of SCI (Hosseini et al., 2016); sodium alginate opioid antagonists complexes encapsulated in macrophage membrane nanovesicles improved motor function and inhibited inflammation in mouse SCI models (Liu et al., 2022b).

Being so, this material shows great potential and promising outcomes to continue to be explored to treat conditions such as SCI, presenting several of interest for this specific application. Although the material demonstrates potential, it remains untested in human clinical settings.

### Agarose

Like alginate, agarose is a natural linear polysaccharide, extracted from red algae, consisting of two residues: β-1,3 linked D-galactose and α-1,4 linked 3,6-anhydro-αL-galactose (Normand et al., 2000). This material presents excellent biocompatibility, which, coupled with unique physicochemical features such as its crosslinker-free gelation process, makes it a promising material for tissue engineering applications, namely for cell growth and controlled drug delivery systems (Zarrintaj et al., 2018).

Agarose-based biomaterials present a biomimetic environment, providing a three-dimensional habitat with minimal immune rejection potential and controlled permeability to oxygen and nutrients. Its potential for morphogenesis derives from several properties, including similarities to the ECM, nonimmunological characteristic, low cost, and appropriate mechanical qualities (Zarrintaj et al., 2018). Agarose-based biomaterials usually take the form of scaffolds, developed through processes such as cell-encapsulating constructs, hydrogels, fibers, or 3D printing; these can be combined with other materials to enhance its performance (Zarrintaj et al., 2018).

In the context of SCI, agarose scaffolds seeded with bone marrow stromal cells expressing the neurotrophin-3 growth factor have been described to significantly improve the organization and extent over which long-tract axons regrow through the lesion site, nonetheless not re-entering the host spinal cord outside of the lesion location (Gros et al., 2010). Implantation of a Matrigel-loaded agarose scaffold resulted in enhanced patient mobility, accompanied by increased cell proliferation and stimulated axonal regeneration and reconnection in a rat model of injury (Han et al., 2018a). Another study showed that an agarose-gelatin-polypyrrole (PPy) hydrogel, with similar mechanical properties to the spinal cord, was able to cover tissue defects and cavities, stimulate neural stem cell differentiation, inhibit astrocyte formation, and promote nerve regeneration, improving recovery from injury (Yang et al., 2022).

Besides the properties this material presents, its biggest limitations for SCI treatment seem to be the necessity to use it in combination with other compounds, such as growth factors, to improve regeneration extension. No materials with agarose have been used in clinical trials yet.

### Chitosan

Chitosan is one of the most plentiful natural materials, found in large quantities in insect and crustacean exoskeletons and shells (Harutyunyan and Lasareva, 2023). Produced by deacetylating chitin, physicochemical properties of this polymer are primarily determined by several parameters such as crystalline structure, degree of deacetylation, and molecular weight (Xiang et al., 2023).

The molecular structure of chitosan is similar to that of glycosaminoglycan, the main component of the ECM; this feature results in low immunogenicity and good biocompatibility for tissue engineering, manifested by good affinity for cell growth (Xiang et al., 2023). Chitosan and its derivates can exist as fibers, gels, films, granules, and nanoparticles, with unique properties such as biocompatibility, biodegradability, non-toxicity (Cho et al., 2010; Harutyunyan and Lasareva, 2023), antibacterial activity, and ability to adsorb metals, making it an interesting material that attracted great attention in the field of nerve tissue repair (Zhang et al., 2019). Injectable chitosan nanoparticles may heal and seal damaged biological membranes, reducing the symptoms of acute SCI (Xiang et al., 2023). However, this polymeric material presents some limiting factors, such as poor solubility or low surface area (Harutyunyan and Lasareva, 2023).

Its neuroprotection and physiological recovery were tested in a compression injury model, showing increased sealing of damaged nerve cell membranes and restoring impulse conduction along the spinal cord (Cho et al., 2010). Implementation of fragmented physical hydrogel suspensions containing chitosan and water as fragments in a rat SCI model showed promising results in reducing glial scarring, promoting vascular and spinal reconstruction, and modulating astrocyte and inflammatory responses, which, together with its safety and lack of host immunogenic responses are argued to make a viable SCI treatment option (Chedly et al., 2017). These reasons, allied to its versatility, make chitosan an auspicious material to develop novel treatments for SCI recovery. Currently, the material is limited to laboratory research and has not entered the clinical trial phase.

### Collagen

Besides dynamic signaling messages controlling cell activity and tissue morphogenesis, providing structural support is a crucial role of ECM proteins; of these, the fibroblast-produced collagen is the most prevalent in the human body (Sorushanova et al., 2019). Biocompatible and biodegradable, it grants mechanical integrity, allowing tissues to maintain three-dimensional stability and form, particularly relevant in the context of a compact beam structure such as the spinal cord. Collagen also activates mechanotransduction pathways within cells, governing several biological functions, including migration, differentiation, angiogenesis, and proliferation, promoting wound healing (Huang et al., 2023).

In the tissue engineering field, it is valued due to its cell recognition signals, capacity to produce 3D scaffolds with different conformations, controllable mechanical properties, and biodegradability (Sorushanova et al., 2019). However, isolated natural collagen presents poor mechanical strength, reduced thermal stability, and enzyme resistance. These are, however, optimized by chemical, physical, and enzymatic crosslinking methods (Gu et al., 2019). Crosslinking collagen increases mechanical stability, degradation rate, and modulates immunogenicity of the implanted scaffold. A collagen-binding vascular endothelial growth factor scaffold promoted axonal regeneration and neovascularization in a SCI model in rats (Wang et al., 2018b), while a N-calmodulin-modified linearly ordered collagen scaffold (LOCS) enhanced mechanical adherence of endogenous neural/progenitor stem cells, resulting in neuro-regeneration and motor recovery in a similar model (Liu et al., 2020a). In a different study, using SCI-transacted canines, the implantation of LOCS with human placenta-derived mesenchymal stem cells (MSCs) improved neural regeneration and functionality, promoting remyelination and synapse formation (Han et al., 2018b).

Collagen has been successfully applied in the form of a scaffold, showing great results promoting regeneration and functional recovery, but more research is needed to develop and apply this material in other forms. NeuroRegen scaffolds have been used in clinical trials with patients who suffer from complete SCI at thoracic and cervical levels (ClinicalTrials ID NCT02510365). Combined with autologous bone marrow mononuclear cells, in a 3-year period, these scaffolds were able to improve the sensory function of the thoracic SCI patients, but no motor recovery was observed (Chen et al., 2020). Conversely, 1 year after implanting these NeuroRegen scaffolds with human umbilical cord MSCs, patients with both cervical and thoracic lesions experienced sensory and motor functional recovery (Xiao et al., 2018). This material has also been combined with epidural electrical stimulation for clinical trials, and results have not yet been made public (ClinicalTrials ID NCT03966794).

### Gelatin

The polypeptide gelatin derives from partially hydrolysed natural collagen (El-Seedi et al., 2023). Gelatin is a biocompatible and biodegradable material with low antigenicity and toxicity, with interesting properties, namely the capacity to promote cell adhesion, proliferation, and differentiation (Ndlovu et al., 2021). Inexpensive to isolate, it has been used in a variety of tissues (including neural), from wound dressing or hydrogels for controlled release of therapeutic agents, to microparticles for tissue regeneration (Lukin et al., 2022).

Due to its quick disintegration period and hydrophilic surface, gelatin is not a good foundation material for wound dressings (Ndlovu et al., 2021), but it is nonetheless widely applied mixed with other polymers to create adhesive, conductive, self-healing, or self-repairing hydrogels (Lukin et al., 2022).

A hybrid alginate/gelatin hydrogel scaffold loaded with PLGA nanoparticles encapsulating the antioxidant curcumin and human endometrial stem cells promoted spinal regeneration in rats (Ai et al., 2023), while a 3D bioprinted alginate/gelatin scaffold was found to be an effective cell transplant carrier for NSCs and oligodendrocytes, improving healing, regeneration, and motor function in complete transected spinal cord rat model (Liu et al., 2022a).

This material is frequently used as the 3D hydrogel gelatin methacryloyl (GelMA): bone MSCs and NSCs added to a 3D GelMA and implanted in a rat model, considerably reduced scarring and inflammation, while simultaneously enhancing neuronal differentiation and motor function (Zhou et al., 2020); combining GelMA 3D hydrogels with induced pluripotent stem cell-derived NSCs increased NSC proliferation and differentiation, decreasing inflammation and promoting tissue regeneration in a complete transaction spinal cord rat model (Fan et al., 2018). More recently, a supramolecular bioink containing GelMA loaded with NSCs and a O-GlcNAc transferase inhibitor small molecule (OSMI-4) promoted neuronal regeneration, axonal growth, and SC repair in rats (Liu et al., 2022c).

Gelatin has been used to encapsulate an antagonist of voltage-gated potassium channels named 4-aminopyridine to be tested in a clinical setting that used several patients with paraplegia and tetraplegia after complete SCI (ClinicalTrials ID NCT03899584) but no results have been published regarding its safety or regeneration capacity.

### Fibrin

Fibrin is a biocompatible and easily manipulated protein polymer. It results from fibrinogen polymerization via serine protease thrombin activation in response to damage, thus being a fundamental component of the coagulation cascade (Brown and Barker, 2014). Besides hemostasis and wound healing, fibrin is essential for physiological and pathological processes such as adhesion, migration, proliferation, differentiation, wound healing, angiogenesis, and inflammation (Litvinov and Weisel, 2017).

As a viscoelastic polymer, its most notable rheological characteristic is its remarkable elasticity (Litvinov and Weisel, 2017), and viscosity (Weisel and Litvinov, 2017) which, allied to hemostatic and regenerative functions, quick polymerization dynamics, ease of tunability, and biocompatibility, explain its wide use as a scaffold in tissue engineering and drug delivery applications (Brown and Barker, 2014), particularly as molecule- and cell-carrying hydrogels (Yu et al., 2020).

A fibrin hydrogel nerve conduit loaded with Wnt5a (an axonal growth-promoting protein kinase C) induced regeneration in an *in vivo* nerve injury model (Liu et al., 2022d). Fibrin has also been combined with natural and synthetic polymers, namely carbon microfibers, in a porcine SCI model, where it promoted axonal growth, elongation, and alignment, but failed to prevent fibrosis (Alves-Sampaio et al., 2023). In a mouse model, a similar composite established that carbon microfiber increased long-term biocompatibility, slowing down polymeric degradation, increasing axonal regeneration, and immune cells mobilisation, increasing the anti-inflammatory response (Escarrat et al., 2023).

In a recent study, fibrin, alone or combined with human induced NSCs, promoted motor recovery in a rat transection SCI model, improved by induced NSC combination, with both approaches inhibiting the secondary injury-associated inflammatory cascades (Liu et al., 2024a). Additionally, fibrin has started to be explored as a biocompatible glue, but it has so far failed to significantly improve functional recovery (Zabbia et al., 2024).

Therefore, fibrin has been explored on its own or in combination with other compounds, to successfully improve function and shows great potential to be further researched in the context of SCI treatment but is yet to be approved for clinical trials.

### Hyaluronic acid

The primary constituents of the ECM are glycosaminoglycan heteropolysaccharides, including the unbranched hyaluronic acid. This linear polymer consists of a repeating unit of β-1,4-D-glucuronic acid-β-1,3-N-acetyl-D-glucosamine (Wei et al., 2024), produced in almost all cell types and present in crucial biological functions, including cellular, such as proliferation and differentiation, or tissue ones, such as lubrication and matrix structure (Marinho et al., 2021). Hyaluronic acid is biocompatible, biodegradable, highly hydrophilic, and is anti-inflammatory and immunomodulator (Marinho et al., 2021), being upregulated during injury, regulating tissue repair (Liang et al., 2016). As such, it is used in clinical applications: wound and tissue repair (Marinho et al., 2021), cell and drug delivery (Burdick and Prestwich, 2011).

The medical field has greatly benefited from hyaluronic acid-based products (Jensen et al., 2020): dopamine-modified and ibuprofen-loaded hyaluronic acid hydrogels were shown to have no toxic effects in neuronal cell lines, while simultaneously directing axonal development, filling up lesion gaps, and transporting bioactive substances (Duarte et al., 2024). A hydrogel was also tested in a rat model, being easily injectable and filling the injury cavity, helping in the healing process, promoting axonal regeneration, angiogenesis, and remyelination (Fan et al., 2024). Selenium-combined hyaluronic acid nanoparticles were found to be neuroprotective at the injury site, where they accumulate, promoting SCI recovery by eliminating reactive oxygen species and suppressing inflammatory response (Luo et al., 2024). Combined with chondroitin sulphate, hyaluronic acid has also been recently investigated for the treatment of urinary tract infections secondary to SCI, with encouraging results (King et al., 2023).

Due to its properties of interest, this natural polymer has been extensively researched, making it a possible candidate to improve results in several clinical areas, such as SCI treatment.

Hyaluronic acid, being a glycosaminoglycan, is one of the main compounds of iAluRil, a medical device that was tested in patients with acute SCI, aiming to prevent urinary tract infections, and this treatment is currently approved for clinical application (ClinicalTrails ID NCT03945110).

**Polyhydroxybutyrate**

PHB is a non-toxic polyhydroxyalkanoate polymer, found in bacterial cytoplasm. PHB is an easy to process, mechanically strong, biodegradable, and biocompatible material that enables support for neuron growth and proliferation (McAdam et al., 2020).

Research evaluating Schwann cells (SCs) viability and morphology on PHB scaffolds revealed cell infiltration and attachment, resulting in a three-dimensional network composed of interlaced fibers and cells, an environment replicating the natural peripheral nervous system ECM (Lezcano et al., 2023).

Poly(hydroxybutyrate-co-hydroxyvalerate) is a copolymer commonly used in tissue engineering due to its biodegradability, biocompatibility, bioactivity, and piezoelectric properties (Pinho et al., 2024). Poly(hydroxybutyrate-co-hydroxyvalerate)/cobalt ferrite microspheres have been found to promote neuronal differentiation and enhance cell viability in *in vitro* reactive oxygen species-associated lesions but also promote microglia activation, which can have either beneficial or harmful effects (Pinho et al., 2024). Several rat models have been used to study PHB/PLA/collagen composites: on one hand, its application as a nanofibrous scaffold was shown to be biocompatible, promoting astrocyte differentiation and inhibiting activation (Zhao et al., 2018); on the other hand, a PHB/PLA/collagen composite membrane reduced glial scar formation, promoting axon development in rat models of SCI by inhibiting inflammatory bodies and modifying macrophage polarization (Zhao et al., 2020).

PHB, particularly combined with other materials, can be extremely useful in neural tissue regeneration, but further research is required to validate its therapeutic potential on its own before it is assessed in human clinical trials.

### Polysialic acid

PSA is a linear, α-2,8 carbohydrate linked to sialic acid, attached to neural cell-adhesion molecule. This biodegradable polysaccharide has been studied as a therapeutic tool for CNS maladies, due to regulating cell adhesion and promoting axonal growth (Zhang et al., 2018). A recent study used rat models of SCI and explored two techniques for modifying SCs with PSA to enhance their effectiveness in SCI treatment, using either a lentiviral vector to introduce the *ST8SIA4* gene or a bacterial enzyme. Both approaches improved SC migration and support for nerve regrowth after SCI transplantation, with PSTNm generating a broader PSA response and neither method triggered a heightened immune reaction, highlighting their potential for advancing nerve regeneration therapies (Pearse et al., 2021).

The efficacy and safety of human polysialylated-neural cell adhesion molecule (PSA-neural cell-adhesion molecule)-positive neural precursor cells were recently found to improve locomotor function following transplantation into rat injured spinal cords (Cleveland Clinic, 2024). By using peptides that functionally mimic PSA had previously been shown to improve SCI recovery, when applied acutely, by increasing plasticity (Mehanna et al., 2010); more recently, a PSA-based minocycline nanodrug delivery system was found to improve motor function, protecting neurons and myelin and recruiting endogenous NSCs to the injury site (Wang et al., 2019).

Although showing interesting results, on its own or modified, PSA has also been explored as a copolymer: a PCL/PSA nanofiber scaffold with glucocorticoid methylprednisolone was used to treat a transected rat spinal cord, seemingly minimizing demyelination and scar formation, promoting axonal growth (Zhang et al., 2018).

Being so, PSA is directly correlated with neural molecules (by mechanisms not yet entirely undisclosed), making it a great polymer for nervous tissue engineering, showing that it can be used in different forms to treat SCI and other diseases that affect the nervous system. However, no clinical trials have been conducted using this polymer to treat SCI patients.

## Synthetic Polymers

Synthetic materials replicate the physicochemical and mechanical characteristics of biological tissues and are frequently employed in tissue engineering and regenerative medicine to treat SCI. Furthermore, synthetic materials are argued to limit the inflammatory response and glial scarring, and present minimal toxicity to the CNS. Additionally, hydrolysis is the route of deterioration for synthetic materials, but this process presents certain disadvantages, such as the release of carbon dioxide, which lowers local pH and can promote cell and tissue necrosis (Liu et al., 2006; Subramanian et al., 2009).

### Polycaprolactone

PCL is a synthetic polymer with good mechanical properties, slow degradation rate, biocompatibility, and low toxicity, allowing an easy conversion into various porous scaffold configurations (Feng et al., 2023; Esmaeili et al., 2024; Javkhlan et al., 2024). Although PCL scaffolds present a low rate of degradation (Shahriari et al., 2017; Javkhlan et al., 2024) and lack active surfaces to promote cell interactions, preventing cell attachment and proliferation (Janmohammadi et al., 2023), PCL has been employed in medical applications, such as tissue engineering scaffolds or drug/gene delivery (Zhang et al., 2019). To improve its performance and applicability, most studies have focused on PCL modification or combination with other materials: scaffolds processed by salt-leaching allow porosity alteration, improving cell adhesion and allowing axonal growth on the open areas, when tested on SCI rat models (Shahriari et al., 2017); coating with Poly-D-Lysine was recently shown to promote cell growth and differentiation of the neural cell line PC12 (Javkhlan et al., 2024); PCL-chitosan scaffolds have been extensively used in tissue engineering for tissue regeneration, including skin, bone, muscle, heart, and nerve (Esmaeili et al., 2024), where results show increased cell proliferation and nerve tissue regeneration along PCL-chitosan fibers (Cooper et al., 2011); PCL-gelatin scaffolds seeded with human endometrial stem cells improved neuronal cell growth in hemisected SCI rats, demonstrating high potential to heal injured tissue (Babaloo et al., 2019).

Being so, although PCL on its own appears to be insufficient for application in the context of SCI and has not yet been used in clinical trials, it is a promising component for combinatory materials.

### Polylactic acid

PLA is a renewable, nontoxic thermoplastic polymer derived from natural resources such as corn starch and sugarcane, approved for medical application by the US Food and Drug Administration, due to its biocompatibility and biodegradability (Li et al., 2020; Ranakoti et al., 2023). Lactic acid is the simplest hydroxy acid and has an optically active asymmetric carbon atom, resulting in two optical isomers: L-lactic acid and D-lactic acid (Li et al., 2020).

Research initially staggered due to its poor strength, low heat resistance, and difficulties machining, but these physical qualities have since been improved by changing the physicochemical properties of PLA, making it a viable material for medicinal applications (Li et al., 2020; Ranakoti et al., 2023). PLA is thus now extensively utilized in the medical field, including tissue engineering, drug delivery, and implants (Li et al., 2020).

Aligned PLA microfiber grafts on their own, without any other pharmacological or cellular tools, have been described to stimulate regeneration in a rat SCI model (Hurtado et al., 2011). Core-shell nanofiber membranes containing PLA shells encapsulated in the omega-3 polyunsaturated fatty acid docosahexaenoic acid were described to increase *in vitro* neurite outgrowth and recovery of loss neurological function in rat models, suggesting its validity as a nanostructured drug delivery device for neuroprotection and neuroplasticity (Liu et al., 2020b).

PLA has been combined with other polymers, such as the neuroprotective iodine-doped plasma pyrrole, and implanted in a rat SCI model: the composite scaffold moderated secondary damage by providing mechanical stability and tissue recovery, and reduced cyst formation, known as a fluid-filled cavity (Osorio-Londoño et al., 2024). This pyrrole combination has shown great potential for SCI, as another study using a PPy/PLA nanofibrous scaffold co-transplanted with bone marrow stromal cells was found to improve recovery in rat SCI, minimizing scar tissue formation, stimulating axon regeneration, and improving electrical conductivity by bridging gaps (Raynald et al., 2019). PLA modifications and its combination with other materials thus show promising results for SCI therapy (see the following subsection).

### Poly(lactic-co-glycolic) acid

PLGA is a degradable organic polymer with interesting properties for therapeutic implantation, such as slow release, low toxicity, biodegradability, and biocompatibility (Rocha et al., 2022). Its lactide to glycolide ratio can be adjusted, modulating specific properties, such as bending and permeability, making it a very adaptive material for tissue engineering scaffolds and drug and gene delivery (Zhang et al., 2019), and is approved by the US Food and Drug Administration for several human clinical applications (Gwak et al., 2016).

Neurotrophin-3-loaded PLGA carriers, synthesized using NSC and SCs, have shown increased differentiation, synaptic connections, and neurite myelination, *in vitro*, providing a promising experimental basis (Xiong et al., 2012) that needs nonetheless further research using CNS-specific cell types to be validated for transplantation in SCI. A PLGA scaffold with fingolimod, an immunomodulator, was shown to increase SCI rehabilitation by promoting recovery 6 weeks upon distribution locally to the lesion site, i.e., during the acute phase of the injury (Kong et al., 2019). Research also demonstrates increased functional recovery, nerve protection, and remyelination following application of PLGA complex injected with olfactory ensheathing cells in a transected rat model spinal cord (Wang et al., 2018a). Recent work showed regeneration, including remyelination, accompanied by motor recovery, in models treated with PLGA nanoparticles loaded with the anti-inflammatory celastrol (Yin et al., 2023).

PLGA spheres are also used to treat SCI: DC-Chol-modified PLGA nanospheres have been suggested as therapeutic gene delivery vehicles (Gwak et al., 2016), while using PLGA microspheres to deliver rapamycin appears to normalize vasculature and promote axonal regeneration in a rat model (Hakim et al., 2019).

Finally, combining PLGA with poly-L-lactic acid to develop scaffolds for SCI is another promising material to promote spinal cord regeneration after injury: PLGA/poly-L-lactic acid scaffolds containing the phosphodiesterase-4 inhibitor rolipram upregulated axon growth, promoted angiogenesis, and improved hindlimb function three weeks after injury, in a rat model (Zhu et al., 2010). This composite scaffold was also embedded with human oral mucosa stem cells and implanted in a rat model, increasing axonal myelination and neural precursors, while reducing glial scar tissue (Ganz et al., 2017).

In sum, PLGA is a promising polymer, particularly when combined with other materials, such as PLA, to improve its performance.

A clinical trial using a PLGA with poly-L-lysine scaffold, called Neuro-Spinal Scaffold, was performed to assess its safety and clinical evidence using thoracic SCI patients’ treatment. However, the primary endpoint was not met (ClinicalTrials ID NCT03762655).

### Polyethylene glycol

PEG is a non-toxic, non-ionic, linear water-soluble polymer, synthesized from ethylene oxide hydrolysis (Lu et al., 2018; Feng et al., 2023). PEG exhibits different physical properties depending on its molecular weight, and it is used in several tissue engineering applications, from membranes to 3D scaffolds and hydrogels, namely in treating SCI, by being a biocompatible material that inhibits free radical formation and prevents lipid peroxidation (Luo et al., 2002; Lu et al., 2018).

Guinea-pig models are already well-established for this study, and PEG administered in midthoracic region compression injuries has been shown to promote behavioral recovery and reduce damage size (Shi, 2013). *In situ* application of PEG in a spinal transection rat model revealed postoperative healing and removal of the transection gap (Ren et al., 2017), and was recently demonstrated to reduce vascular damage and promote motor recovery in a similar model, suggested to be acting via secondary injury minimization (Lee et al., 2023). PEG has been explored in several other animal models, from mice, where PEG-grafted graphene oxide sheets stimulated tissue repair and inhibited lesion progression in an injury model (Yari-Ilkhchi et al., 2021), to more advanced animal models, such as canines, where it promoted neuroprotection and fiber regrowth (Ren et al., 2019). PEG can be implanted in different forms, showing always promising results in minimizing lesion outcomes and promoting recovery, being one of the most used synthetic polymers for tissue engineering. Nonetheless, acute administration, immediately after injury, consistently appears to be more effective in improving recovery, hypothesized to be due to fibers passing through the fusion contact, bridging the two endpoints before glial scar is formed (Ren et al., 2017, 2019).

PEG-chitosan (Neuro-PEG) has successfully promoted motor recovery in a pre-clinical trial using a swine model of spinal cord transection, and clinical trials are anticipated soon (Lebenstein-Gumovski et al., 2023).

### Poly(2-hydroxyethyl methacrylate)

The flexible and hydrophilic pHEMA is a porous non-degradable acrylic polymer that has been explored in spinal cord recovery in hydrogel form, due to its architectural morphology and mechanical performance (Zhang et al., 2019).

A study investigated the effects of a positive charged co-monomer pHEMA hydrogel seeded with induced pluripotent stem cell-derived neural progenitors in a rat model of SCI. Researchers found that the hydrogel supported the survival and integration of induced pluripotent stem cell-derived neural progenitors into the injured tissue, promoting the growth of host axons and blood vessels, but, despite these positive interactions, there was no significant improvement in locomotor function observed in the treated animals (Ruzicka et al., 2019). A synthetic hydrogel channel composed of poly (2-hydroxyethyl methacrylate-co-methyl methacrylate) (pHEMA-MMA), that facilitates motor axon regeneration (Tsai et al., 2004), significantly increased axonal regeneration and functional recovery in a rat model of SCI, seemingly increasing the density of nerve fibers on NF-200 and CGRP axons (Tsai et al., 2006). A positively charged 2-hydroxyethyl methacrylate (HEMA) hydrogel was implanted in a rat model immediately and 1 week after injury, and immediately after implantation was shown to allow SCs, blood vessels, and neurofilaments to penetrate the structure and bridge spinal cord stumps; implantation delay by a week resulted in a bigger decrease of the posttraumatic cavity volume, showing the implantation of a bridging scaffold during acute phase may not be advantageous (Hejcl et al., 2008). Finally, pHEMA scaffolds modified with the laminin-derived SIKVAV promote cell adhesion and growth and, when transplanted into hemisected and transected spinal cords of rat models, facilitated tissue bridging and oriented axonal growth, with porosity- and elasticity modulus-dependent effects (Kubinová et al., 2015). A more recent study on SIKVAV-modified pHEMA hydrogels found that while connective tissue and blood vessels infiltrated rapidly, axonal growth occurred gradually over a month but was not enhanced by MSC seeding (Hejčl et al., 2018). A scaffold of pHEMA-incorporated collagen is described to promote blood vessel regeneration and axonal penetration into the scaffold (Giannetti et al., 2001).

Despite its potential, pHEMA is underutilized in recent studies on neural tissue engineering, necessitating further modifications to enhance sustained regeneration and functional recovery, and is so yet to be explored in clinical trials. This presents significant opportunities for ongoing research and innovation in tissue engineering.

### Poly N-(2-hydroxypropyl) methacrylamide

pHPMA is another non-degradable acrylic polymer that, particularly in the form of a hydrogel, has been explored for SCI since it is more biocompatible than pHEMA.

A functionalized pHPMA, modified with a cell-adhesive sequence-containing synthetic peptide, presented physical properties (such as viscoelasticity and conductivity) comparable to those of central nervous system tissue, when implemented on an injured adult rat spinal cord model, promoting neurogenesis and tissue regeneration (Woerly et al., 2001). One pHPMA derivative, called NeuroGel, was found to swell in aqueous media, preserving a stable chemical and physiological environment, crucial for cell growth in nerve tissue. It was thus combined with hippocampus NSCs and shown to inhibit glial development and promote neuron differentiation, *in vitro*, resulting in *in vivo* reduction of spasticity and recovery in a rat model (Rybachuk et al., 2023).

However, few recent studies have explored the use of this synthetic polymer for SCI treatment, and it is thus far from being explored in humans.

### Polyurethane

Polyisocyanates, polyols, and auxiliary agents react to form the versatile polymeric material polyurethane (PU) (Dang et al., 2024). PU is extensively explored in tissue engineering due to its biocompatibility, biodegradability (slower than any natural polymer) and tunable mechanical properties, offering remarkable adaptability and healing potential, making it a valued tool in various biomedical fields, such as drug delivery systems and wound healing (Lin et al., 2020; Wang et al., 2024).

A 3D scaffold made of biodegradable waterborne PU (a coating and adhesive technology that uses urethane dissolved in water particles) was tested on the fibroblast-like L929 cell line to promote both oriented cell growth and anisotropic tissue regeneration, suggesting a potential to promote biological anisotropic tissue regeneration in the spinal cord (Lin et al., 2020). In a rat model of peripheral nerve injury, a study introduced a wireless, bioresorbable stimulator made from dynamic covalent PU to support nerve regeneration, showing that targeted electrical stimulation reduced muscle degeneration and enhanced recovery (Choi et al., 2020).

Due to its characteristics, PU is commonly combined with other polymers to improve its properties and performance. Neuro2a cells were shown to grow and align along porous nerve guidance channels, generated using PU-based nerve conduits with lumenal chitosan-collagen, resulting in electrophysiological and morphological improvements in *in vivo* peripheral injury models (Singh et al., 2021). Nerve guidance channels were also found to increase macrophagic recruitment and promote SCs migration and myelination in a rat model of sciatic nerve injury, indicating a potential for peripheral nervous system regeneration, but the complexity of the central nervous system requires a more systematic approach (Sun et al., 2024). Indeed, PU-polylactide composite sponges demonstrate promising outcomes in fibroblast cultures, such as great porosity, flexibility, and degradability (Lis-Bartos et al., 2020). In a rat spinal cord contusion model, a PU-based reverse thermal gel demonstrated a potential protective effect on transplanted bone marrow stromal cells, resulting in behavioral gains (Ritfeld et al., 2014).

Even though PU shows great potential to be used for SCI recovery research, most published work using this polymer to study regeneration is focused on the peripheral nervous system, so a need remains unmet regarding the application of this polymer for this specific application.

### Polyvinyl alcohol

In addition to high strength and resistance to deformation, water retention, and porous structure, PVA has been explored for scaffold application, particularly due to its anti-inflammatory and protective properties (Nathan et al., 2023). Scaffolds based on the PVA-chitosan have been shown to provide an appropriate structure for tissue regeneration (Dashtdar et al., 2015) and a previous study had developed electrospun PVA/chitosan nanofibrous scaffolds for nervous tissue engineering, demonstrating that adding chitosan increases nerve cell viability and proliferation, improving biocompatibility (Alhosseini et al., 2012). From *in vitro* and *in vivo* experiments, PVA-PVP thin films may efficiently transport inhibitors to p38 and JNK following SCI. Tissue histology validated the film ability to compress tissue and release medicines, indicating neuroprotective properties and the potential to reduce progressive cell death (Comolli et al., 2012). PVA nerve conduits (obtained by coupling PVA with ECM proteins and fucoidan to increase cell attachment, as PVA lacks cell-binding sites) were employed to enhance neurogenesis and axon guidance in PC12 cells, improving neurite outgrowth and axon guidance that are considered decisive steps in nerve regeneration and, consequently, functional recovery (Yao et al., 2023). A composite molybdenum sulfide/graphene oxide/PVA hydrogel showed potential to stimulate neuron differentiation, limit inflammation, inhibit glial cell activation, and restore motor function, albeit with moderate mechanical characteristics (Chen et al., 2022).

Combining desirable mechanical properties with an anti-inflammatory effect, PVA is thus a promising synthetic material, anticipated to continue to be explored for tissue engineering applications during the next decade, presumably eventually making its way to pre-clinical trials.

The pros and cons of each natural and synthetic polymer are summarized in **Table 3**.

**Table 3:** Summary of the best characteristics and limitations of each polymer to be used for SCI treatment, based on described studies

Polymers	Best characteristics	Limitations	References
**Natural polymer**			
Alginate	Biodegradable, biocompatible, non-toxic and easy to obtain. Forms gels. Supports tissue engineering, wound healing, drug delivery, and cell culture. Can encapsulate drugs and cells.	Biological behavior depends on composition, administration, and external factors. Requires optimization for specific applications such as SCI treatment.	Lee and Mooney, 2012; Hosseini et al., 2016; Grijalvo et al., 2019; Zhang et al., 2021; Liu et al., 2022b; Adamiak and Sionkowska, 2023
Agarose	Biocompatible and mechanically stable. Provides a biomimetic 3D environment for cell growth. Can be processed into several forms. Supports axonal regeneration and neural tissue repair	May not sufficiently support SCI treatment. Often requires a combination with other materials to enhance performance.	Gros et al., 2010; Han, et al., 2018a; Zarrintaj et al., 2018; Yang et al., 2022
Chitosan	Biodegradable, biocompatible, and non-toxic. Similar to ECM glycosaminoglycan, making it ideal for neural tissue engineering. Can exist in various forms. Promotes sealing of nerve membranes, spinal cord reconstruction, and nerve protection	Poor solubility and low surface.	Cho et al., 2010; Chedly et al., 2017; Zhang et al., 2019; Harutyunyan and Lasareva, 2023; Xiang et al., 2023
Collagen	Biodegradable, biocompatible, and promotes wound healing. Provides structural and mechanical support. Used in 3D scaffolds with tunable properties. Encourages cell differentiation, angiogenesis, and tissue repair. Enhances axonal regeneration and neural functional recovery.	Poor mechanical strength and thermal stability. Requires crosslinking for improved mechanical properties and degradation control. Limited exploration in other formats.	Han et al., 2018b; Wang et al., 2018b; Gu et al., 2019; Sorushanova et al., 2019; Liu et al., 2020a; Huang et al., 2023
Gelatin	Biocompatible, biodegradable, and low-cost. Promotes cell adhesion, proliferation, and differentiation. Used in wound dressings, hydrogels, and microparticles for tissue regeneration. Commonly applied as GelMA. Enhances neural regeneration and functional recovery.	Not suitable as a standalone wound dressing due to rapid degradation. Combining it with other materials improves its mechanical stability	Fan et al., 2018; Zhou et al., 2020; Ndlovu et al., 2021; Liu et al., 2022a, c; Lukin et al., 2022; Ai et al., 2023
Fibrin	Biocompatible. Good mechanical stability and flexibility. Supports angiogenesis, cell adhesion, proliferation, and differentiation. Allows molecule and cell delivery. Enhances axonal regeneration and motor functional recovery.	May not prevent fibrosis, which can impede functional recovery. Works best when combined with other materials	Brown and Barker, 2014; Litvinov and Weisel, 2017; Weisel and Litvinov, 2017; Yu et al., 2020; Liu et al., 2022d; Alves-Sampaio et al., 2023
Hyaluronic Acid	High hydrophilicity, biocompatibility, and biodegradability. Regulates tissue repair and inflammation. Supports cellular proliferation, differentiation, and morphogenesis. Injectable hydrogels promote axonal regeneration and remyelination. Can be used for cell/drug delivery	Often used in combination with other materials for enhanced effects.	Burdick and Prestwich, 2011; Liang et al., 2016; Jensen et al., 2020; Marinho et al., 2021; King et al., 2023; Duarte et al., 2024; Fan et al., 2024; Luo et al., 2024
PHB	Biodegradable, biocompatible, and non-toxic. Strong mechanical properties. Supports neuron growth and proliferation. Copolymer shows additional bioactivity. Enhances astrocyte differentiation and inhibits excessive activation	Mostly used in combination with other polymers.	Zhao et al., 2018, 2020; McAdam et al., 2020; Lezcano et al., 2023; Pinho et al., 2024
PSA	Natural polysaccharide critical for nervous system development. Regulates cell adhesion and promotes axonal growth. Supports Schwann cell migration and neurorepair. Improves motor functional recovery when combined with neural precursor cells.	Used in combination with other materials. Often requires genetic or enzymatic modifications. Better for drug delivery systems or scaffold-based approaches.	Mehanna et al., 2010; Zhang et al., 2018; Wang et al., 2019; Pearse et al., 2021; Cleveland Clinic, 2024
**Synthetic polymer**			
PCL	Biocompatible and low toxicity. Slow degradation rate allows for long-term support. Easily processed into porous scaffolds.	Low degradation rate may delay tissue remodeling. Lacks active surfaces for cell attachment and proliferation.	Cooper et al., 2011; Shahriari et al., 2017; Zhang et al., 2019; Babaloo et al., 2019; Feng et al., 2023; Janmohammadi et al., 2023; Esmaeili et al., 2024; Javkhlan et al., 2024
PLA	Renewable, biodegradable, and biocompatible. Good mechanical properties after modification.	Initial poor strength and low heat resistance. Requires a combination with other materials	Hurtado et al., 2011; Raynald et al., 2019; Li et al., 2020; Liu et al., 2020b; Ranakoti et al., 2023; Osorio-Londoño et al., 2024
PLGA	Degradable and biocompatible Tunable lactide-to-glycolide ratio for controlled properties. Suitable for drug and gene delivery.	Short degradation time. Often requires a combination with other materials to enhance functionality.	Zhu et al., 2010; Xiong et al., 2012; Gwak et al., 2016; Ganz et al., 2017; Wang et al., 2018a; Hakim et al., 2019; Kong et al., 2019; Zhang et al., 2019; Rocha et al., 2022; Yin et al., 2023
PEG	Non-toxic, water-soluble and biocompatible. Inhibits free radical formation and lipid peroxidation. Versatile in SCI applications, from scaffolds to hydrogels.	Lacks long-term inherent structural support. Needs modification for enhanced cell adhesion and tissue integration.	Luo et al., 2002; Shi, 2013; Ren et al., 2017, 2019; Lu et al., 2018; Yari-Ilkhchi et al., 2021; Feng et al., 2023; Lee et al., 2023
pHEMA	Hydrophilic, porous, and flexible. Biocompatible and can be used as a hydrogel. Supports axonal regeneration when modified.	Non-degradable, limiting long-term integration. Less commonly studied for SCI.	Giannetti et al., 2001; Tsai et al., 2004, 2006; Hejcl et al., 2008; Kubinová et al., 2015; Hejčl et al., 2018; Ruzicka et al., 2019; Zhang et al., 2019
pHPMA	High biocompatibility. Forms stable hydrogels. Can be functionalized for enhanced neurogenesis.	Non-degradable, requiring adaptation for long-term SCI applications. Limited studies compared to other polymers.	Woerly et al., 2001; Rybachuk et al., 2023
PU	Highly adaptable mechanical properties. Biodegradable and biocompatible. Used in various biomedical fields, including nerve regeneration.	Primarily studied for peripheral nerve injuries. Requires systematic approaches for CNS applications.	Ritfeld et al., 2014; Choi et al., 2020; Lin et al., 2020; Lis-Bartos et al., 2020; Singh et al., 2021; Sun et al., 2024; Wang et al., 2024
PVA	High mechanical strength and water retention. Can be combined with other materials for improved biocompatibility. Anti-inflammatory properties.	Lacks natural cell-binding sites, requiring modification. Moderate mechanical characteristics may limit scaffold performance.	Comolli et al., 2012; Alhosseini et al., 2012; Dashtdar et al., 2015; Chen et al., 2022; Nathan et al., 2023; Yao et al., 2023

ECM: Extracellular matrix; GelMA: gelatin methacryloyl; PCL: polycaprolactone; PEG: polyethylene glycol; PHB: polyhydroxybutyrate; pHEMA: poly(2-hydroxyethyl methacrylate); pHPMA: poly (N-(2-hydroxypropyl) methacrylamide); PLA: polylactic acid; PLGA: poly(lactic-co-glycolic acid); PSA: polysialic acid; PU: polyurethane; PVA: polyvinyl alcohol; SCI: spinal cord injury.

## Emergent Technologies

Most materials used for tissue regeneration, including neural tissue, are non-responsive, which can be a challenge because they are not able to adapt to the environment or actively interact with cells, making it harder to support the complex and dynamic processes related to neural tissue regeneration. However, the creation of smart materials with stimulus/response behavior has recently attracted attention in polymer research. Stimuli are divided into three classes: physical, chemical, and biological. For instance, the most common physical stimuli are temperature, laser light, magnetic field, ultrasound, and mechanical deformation; chemical stimuli include pH, solvent, ionic strength, or electrochemical responses; the usage of specific enzymes and receptors are examples of biological stimuli (Delcea et al., 2011). These stimuli induce chemical modifications and, consequently, changes in the structure of polymeric materials, reversible or irreversible (Zhao et al., 2025). When considering an electric stimulus, the reaction may be caused by either ionic or electric conduction, which can cause an electric current flow, stimulating cells, causing antimicrobial activity, or altering other properties. As CNS functions are based on electrical signaling, and they play a crucial role in recovering the loss function during SCI, electroactive materials have been reported and utilized in drug delivery and neural tissue regeneration (Palza et al., 2019).

Conductive polymers have been commonly used in 3D scaffolds. As they tend to be non-degradable, non-soluble, and mechanically inappropriate (on their own) for SCI treatment, conductive materials are normally combined with others, creating a composite scaffold that presents the main properties of the material, but gains electronic conductivity (Liu et al., 2024b), useful for regenerating neural tissue. PPy, polyaniline, and poly(3,4-ethylenedioxythiophene) are among the most used conductive materials. Incorporating PPy in a gelatin–hyaluronic acid scaffold improved conductivity, mechanical strength, and biocompatibility *in vitro*, highlighting its potential for SCI repair (Serafin et al., 2023), and combining a chondroitin sulfate-gelatin hydrogel extracellular matrix with PPy resulted in a conductive, self-healing, and injectable material, purposed to support axonal regeneration and functional recovery in a rat SCI model (Luo et al., 2022). Polyaniline, added to an electrospun nanofiber mesh, improved conductivity and bioactivity, enabling electrical scaffold stimulation, which enhanced neuronal differentiation and reduced astrocyte formation on *in vitro* tests with rat NSCs and PC12 cells (Yan et al., 2020). The incorporation of poly(3,4-ethylenedioxythiophene) nanoparticles in a gelatin-hyaluronic acid scaffold significantly improved conductivity and biocompatibility, resulting in enhanced neuronal regeneration and reduced inflammatory response around the injury site, in a rat SCI model (Serafin et al., 2022).

All things considered, the fabrication of advanced smart materials by combining non-responsive polymeric matrices with conductive polymer components is a promising innovative approach for neural regeneration and recovery following SCI. This strategy combines the complementary properties of both types of polymers to create a conducive microenvironment supporting neuronal growth and enhancing signal conduction.

Alternatively, translation of novel discoveries from basic science into a therapeutic technology can revolutionize regeneration therapies. For example, the application of genetic tools to correct disease-causing mutations, downregulate genes that contribute to a certain disease, or deliver genes that encode molecules with therapeutic potential (gene therapy) has been explored for decades (Blits et al., 2005), but the emergence of new molecular tools, such as CRISPRCas9, has greatly improved its application for SCI recovery (Cunningham et al., 2023). As technology continues to develop, gene-editing materials that combine the previously described pro-regenerative properties with specifically tailored gene therapy processes could revolutionize the field.

Another promising approach in SCI treatments is cell therapy. Several cell types have been used and tested in SCI clinical trials, and can be divided into stem cells, immune cells, or support cells (Ribeiro et al., 2023). Stem cells are the most common used ones, with several clinical trials ongoing and completed, and include mesenchymal, hematopoietic, and neural types of stem cells (Gao et al., 2020). Clinical trials using MSCs have proven to be safe (Geffner et al., 2008; Pal et al., 2009; Hur et al., 2016) and enable motor and sensory recovery in patients who suffer from SCI (Chen et al., 2020). NSCs also present a big capacity to improve their quality of life by minimizing the effects of the lesion (Shin et al., 2015; Zheng et al., 2022). As cell therapy develops (and technologies such as induced pluripotent stem cells start to make their way into clinical applications), the development of cell-delivering scaffolds, or 3D organoid-like materials, could help accelerate tissue regeneration (and, consequently, patient recovery).

These emerging technologies herald a new era of personalized and regenerative treatment strategies, offering renewed hope for functional recovery and improved quality of life in individuals with spinal cord injury.

## Conclusion

Spinal cord injury is one of the most debilitating modern pathologies, with a reserved medical outlook, due to the absence of regeneration and recovery possibilities. Research is looking for tools to safely promote healing, and promising solutions have been developed in recent years. Among the therapies that are being developed, natural and synthetic polymers for tissue engineering and drug delivery are the most common (and successful) ones, with a wide range of material options.

All natural polymers are considered biocompatible, biodegradable, and non-toxic, making them particularly desirable for biomedical applications, most popularly in the form of hydrogels and scaffolds. Most materials require combination with other compounds (such as growth factors, drugs, or synthetic polymers) to improve their mechanical or biological properties; collagen, gelatin, and fibrin are ECM-based and offer strong tissue regeneration potential; polysaccharides such as hyaluronic acid, chitosan, agarose, and alginate are excellent at cell delivery and neuroprotection; PHB and PSA are bioactive but need to be further optimized for SCI treatment.

Although long-term stability and CNS integration remain challenging, synthetic polymers for SCI treatment feature tunable mechanical properties that make them valuable for tissue engineering. While some, such as PCL and PLA, require modifications to enhance bioactivity, others, such as PLGA, PEG, and pHPMA, are neuroprotective and anti-inflammatory on their own. Nevertheless, their versatility and combinatory potential make them strong candidates for future advancements in neural tissue engineering.

In conclusion, polymeric materials have demonstrated great potential in the treatment of SCI, offering innovative solutions for a condition that remains highly debilitating and in need of advanced therapeutic approaches.

## Data Availability

*Not applicable.*
